# A Case of Cachexia Secondary to Obsessive-Compulsive Disorder

**DOI:** 10.1155/2020/5783191

**Published:** 2020-05-27

**Authors:** Hailey L. Gosnell, Anita S. Kablinger

**Affiliations:** ^1^School of Medicine, Virginia Tech Carilion School of Medicine, Roanoke, VA, USA; ^2^Carilion Department of Psychiatry and Behavioral Medicine, Roanoke, VA, USA

## Abstract

Obsessive-compulsive disorder (OCD), a relatively common psychiatric illness, is diagnosed using DSM-V criteria. Its severity is assessed using the Yale-Brown Obsessive Compulsive Scale (Y-BOCS). Symptoms are broken down into five categories of obsessive-compulsive (O-C) manifestations: contamination/cleaning, symmetry/ordering, taboo thoughts, doubt about harm/checking, and worry about throwing away items that could prove useful or valuable/hoarding. CBT in the form of exposure response therapy (ERP) and/or SSRI/clomipramine administration is the mainstay of treatment. We present a unique OCD case in the nature of obsessions and compulsions, cachexia presentation without anorexia, and history of multiple inpatient psychiatric admissions. Our patient's obsessions focus on eating at specific times, prompting compulsive eating patterns that often result in starvation due to missing timeframes that the patient deems acceptable for eating. His resulting cachexia and eventual worsening of depression to the point of suicidality necessitated multiple inpatient stays and placement at a long-term mental health care facility.

## 1. Introduction

Obsessive-compulsive disorder (OCD) is a relatively common psychiatric illness, affecting 1.2% of the US population [1]. The disorder has two peaks of onset: preadolescence and early adulthood [2]. In preadolescence, males are more commonly affected. In adulthood, the reverse is true [2]. The average age of onset is 19.5 years. The onset of symptoms is usually gradual, and the course of the disease is typically chronic [3].

OCD is diagnosed using DSM-V criteria. Upon diagnosis, severity of the disease is measured using the Y-BOCS [4]. The Y-BOCS can be filled out by the patient or the clinician. In addition to the severity rating scale, a symptom checklist is also included to provide clinicians with data on specific themes of obsessions and/or compulsions applicable to the patient both in the present and prior to presentation.

The Y-BOCS checklist includes over 50 obsessions and compulsions representing the majority of OCD symptoms most commonly noted clinically [5]. However, these symptoms are often broken down into five categories: contamination/cleaning, symmetry/ordering, taboo thoughts, doubt about harm/checking, and worry about throwing away items that could prove useful or valuable/hoarding [6]. Obsessions and compulsions are often consistent over time and, if they evolved, would stay in the same category. Alternate presentations may demonstrate changes in themes of both anxiety-provoking thoughts or images and compulsive acts. All categories except taboo thinking respond to current treatment mainstays readily [7].

Cognitive behavioral therapy in the form of exposure response prevention (ERP) with or without adjunct SSRI administration is the most effective treatment to date for OCD [8]. The severity of the patient's illness ultimately determines whether CBT is used alone or in tandem with medication. In the cases of mild to moderate illness severity (a Y-BOCS score of 8 to 23), CBT can be used alone. Any Y-BOCS score above 23 (severe OCD) warrants use of both ERP and medication. In fact, patients with severe OCD often need symptom relief from medication prior to engaging in ERP treatment [8].

ERP treatment is carried out by first educating the patient on the pathophysiology of their illness and the process of ERP. The patient next fills out a log of symptom triggers and their severities rated on a scale of 0 to 100 [9]. Patients are next instructed to work on exposing themselves to situations that will provoke their lowest rated symptom triggers (eventually working up to higher rated symptom triggers). They are encouraged to continue the exposure to each symptom trigger until the anxiety associated with that trigger is decreased substantially [9]. Patients are meant to expose themselves to triggers during therapy sessions as well as at home in order to gain the maximum benefit from ERP. ERP is also often augmented with medication therapy in severe OCD cases.

The FDA has approved five (serotonergic) medications in the treatment of OCD: four SSRIs (fluoxetine, fluvoxamine, paroxetine, and sertraline) and one tricyclic antidepressant (clomipramine). SSRIs are considered the first-line medication therapy for OCD as they carry a lesser side effect burden than clomipramine, with the most commonly reported side effects being apathy, weight gain, and sexual dysfunction [10]. Suicidal ideation in children is another reported side effect of SSRIs, though rare [11].

In those patients who do not respond to a 12-week trial of moderate-dose SSRI therapy, defined as a less than 25% reduction in the Y-BOCS score, clomipramine is next considered. Clomipramine has been shown to be a more effective treatment for OCD, though, as previously stated, its side effect profile is less favorable [12]. Clomipramine causes sedation, weight gain (more severe than that noted in SSRI treatment), and QT interval prolongation [13]. It is important to recognize two other unique characteristics of OCD: that OCD has a very poor response to placebo and is difficult to treat into full sustained remission, both emphasizing that aggressive and long-term treatment is necessary.

Extremely severe cases of OCD that fail to respond to multiple therapeutic interventions can be effectively treated with gamma knife radiosurgery and deep brain stimulation. Gamma knife radiosurgery consists of anterior capsulotomy, limbic leucotomy, and cingulotomy [10]. Deep brain stimulation is initiated via device implantation to augment signaling of the nucleus accumbens, ventral internal capsule, and ventral striatum [14].

## 2. Case Presentation

We present an atypical case of OCD with regard to compulsions resulting in cachexia and need for multiple psychiatric hospitalizations culminating in placement at a long-term mental health care facility. The patient, an 18-year-old male with past medical history of major depression and social anxiety disorder, was brought to the emergency department by his father out of concern regarding his cachectic state as a result of worsening OCD.

The patient reported that he has had OCD symptoms since the age of 12. His obsessions started out as fear of contamination and resulted in excessive washing. His obsessions are now order driven, focused on the need to perform certain rituals (namely, eating) at specific times. He would attempt to carry out compulsive rituals to ensure he could eat at the proper time to mitigate anxiety produced by his obsessions. If the appropriate time to eat passed, the patient would not eat and would have to wait for the next scheduled time. He also reported feeling the need to tap his fingers or stomp his feet a certain number of times before and after performing any activity of daily life.

The patient reports his symptoms have worsened since starting college, primarily because his college schedule does not permit him to eat his meals at the “right times.” He spends the vast majority of his day obsessing over eating at specific times. He reports that his OCD symptoms are preventing him from doing well at school and maintaining a healthy weight. The patient denied anorexia, binging, suicidal ideation, or homicidal ideation.

The patient was offered medications and a psychotherapeutic milieu for his condition, but declined both options, stating that he was determined to fix this issue on his own. Due to the patient's poor insight and extremely low BMI (15.8 kg/m^2^), the medical team decided to admit him to an inpatient psychiatric facility.

Over the course of his four-day inpatient stay, the patient was treated with fluvoxamine. His OCD symptoms decreased in frequency and severity. He reported eating his meals as well as additional snacks throughout each day, gaining five pounds. The patient declined treatment with medication upon discharge and was thus advised to outpatient follow-up.

One week later, the patient returned to the ED with worsening OCD symptoms and suicidal ideation. He was unable to attend his therapy appointment because it interfered with his class scheduling and did not get a chance to reschedule. He had a personal goal of stopping his compulsions but failed to do so. This resulted in extreme guilt and worsened depression, culminating in suicidal thoughts with a plan to ingest a harmful substance. He was also noted to have lost the five pounds he gained during his first inpatient admission due to the fact that he only ate one meal during the week posthospitalization. He was readmitted to the inpatient unit after expressing a desire to gain weight and get his OCD symptoms under control.

His second admission, lasting one week, saw similar progress to the first admission. The patient was restarted on fluvoxamine, resulting in decreased frequency and severity of his obsessions and compulsions. He was able to eat three meals a day and have snacks in between. He gained ten pounds during his admission. The patient was counseled on the importance of taking his medication and attending scheduled outpatient follow-up appointments upon discharge.

Four weeks after the patient's second discharge from the inpatient psych unit, he presented once again to the ED with worsening OCD symptoms and suicidal ideation. He reported attending scheduled follow-up appointments but stopping his medications the day after his second discharge because he was curious about whether he needed the medication. He again attempted to resolve his obsessions and compulsions on his own. His inability to overcome his obsessions and compulsions again caused worsening depression with suicidal ideation. The patient was noted to have lost the ten pounds he had gained during his second inpatient admission. He was admitted into inpatient psychiatry for a third time.

The patient's last admission (duration of sixteen days) saw similar progress to previous admissions. The patient was again started on fluvoxamine. He resumed eating three meals a day with snacks in between, gaining four pounds during his admission. He again reported that his obsessions and compulsions decreased in severity. Halfway through his admission, he scored a 23 on an administered Y-BOCS (indicative of a case on the borderline between moderate and severe). Arguably, this test should have been administered prior to the start of treatment to accurately measure the initial severity of the patient's case as well as the impact of his inpatient stay. Based on his qualitative improvement during the course of his admissions, it is likely the patient would have scored several points higher on the Y-BOCS if administered prior to his first hospitalization. Following discharge, the patient has continued his medication regimen and is pursuing combined ERP treatment in a long-term care facility.

## 3. Discussion

A number of elements made this patient's case of OCD noteworthy including the nature of the patient's obsessions and compulsions, his resulting cachexia without anorexia, and his need for multiple inpatient admissions with subsequent referral to a long-term mental health care facility. The patient's need to perform activities of daily living (namely, eating) at the right time with the right mindset as a result of order-driven obsessive ruminations is not a common compulsive manifestation of OCD. The impact of the patient's symptoms on his quality of life was remarkable, but the resulting sequelae made this condition life-threatening on multiple fronts.

Inpatient hospitalization is atypical for an OCD presentation, let alone multiple inpatient hospital stays and subsequent placement in a long-term facility. However, our patient's symptomology warranted an inpatient stay initially because it resulted in cachexia. During his first admission to the inpatient unit, he was able to gain weight after being stabilized by medication. However, as a result of his resistance to using medication and outpatient ERP, he was unable to maintain the progress he made during his first inpatient stay. His mental state over what he perceived as failure to control his condition on his own caused worsening depression to the point of suicidal ideation with a plan. The two subsequent inpatient stays endeavored and ultimately succeeded in addressing both the patient's cachexia and his severe depression.

The patient's cachectic frame and severe depression taken with his OCD prompt consideration for possible comorbid anorexia. In fact, the comorbidity rate for OCD and anorexia nervosa is reported to be between 10 and 40% [15]. While it is a reasonable differential for the patient presented in this case, a number of elements make this diagnosis less likely. The patient's refusal to eat is a compulsion in response to his obsessions about carrying on activities of daily living at specific times and being in the right mindset. It is not secondary to a desire for weight loss or a distorted body image. The patient is aware of and dissatisfied with his thin frame, a finding that runs contrary to a typical anorexia presentation.

## 4. Conclusion

The nature of our patient's obsessions and compulsions, his resulting cachexia without anorexia, and his need for multiple inpatient admissions make this case remarkable. The patient's fixation with eating at certain times with a specific mindset and his compulsions to suit these obsessions manifest in an unusual OCD picture (and may even have a delusional quality to it?). His need for one inpatient admission, let alone three, to manage his condition is also noteworthy, though it is reasonable considering the severity of his cachexia and depression.
